# The dose of the normal saline pre-infusion and other risk factors for amphotericin B deoxycholate-associated acute kidney injury

**DOI:** 10.2478/abm-2023-0071

**Published:** 2023-12-28

**Authors:** Mathurot Virojanawat, Somkanya Tungsanga, Leilani Paitoonpong, Pisut Katavetin

**Affiliations:** Department of Medicine, King Chulalongkorn Memorial Hospital, Faculty of Medicine, Chulalongkorn University, Bangkok 10330, Thailand

**Keywords:** acute kidney injury, amphotericin B deoxycholate, normal saline, pre-infusion, nephrotoxicity

## Abstract

**Background:**

Conventional amphotericin B deoxycholate (AmBd) is the preferred amphotericin B formulation in countries with limited resources despite its nephrotoxicity. Normal saline pre-infusion is a recommended measure to reduce the risk of nephrotoxicity in patients receiving AmBd.

**Objectives:**

To examine the effect of different normal saline solution (NSS) pre-infusion doses, and other potential risk factors, on the development of acute kidney injury (AKI) in patients with invasive fungal infection receiving AmBd.

**Methods:**

Adult patients with invasive fungal infections who received intravenous AmBd were included in this retrospective study. Doses of the normal saline pre-infusion were adjusted to the body weight (NSS/BW) and the daily dose of amphotericin B (NSS/AmBd). Kaplan–Meier survival analysis was used to estimate 14 d AKI-free survival rates, and the log-rank test was used to compare AKI-free survivals between groups.

**Results:**

The present study included 60 patients; 31 patients developed AKI during the AmBd therapy. The overall 14 d AKI-free survival was 48.3%. NSS/AmBd, but not NSS/BW, was associated with AKI-free survival in patients receiving AmBd: the higher the NSS/AmBd, the higher the AKI-free survival. Gender, baseline blood urea nitrogen (BUN), and baseline plasma bicarbonate (Bicarb) also affected AKI-free survival. Female gender, higher BUN, and lower Bicarb were associated with higher AKI-free survival.

**Conclusions:**

The present study suggests that low NSS/AmBd, male gender, low BUN, and high Bicarb are risk factors for AmBd-associated AKI. Excluding gender, these risk factors are potentially modifiable and would guide tailoring appropriate preventive measures for AmBd-associated AKI.

Amphotericin B is the principal drug for the treatment of life-threatening fungal infections despite its nephrotoxicity risk [1]. Although amphotericin B formulations with low-nephrotoxicity risk are available, conventional amphotericin B deoxycholate (AmBd) is still the preferred formulation when the cost is an issue, especially in developing countries.

Salt loading is a recommended measure to reduce the risk of nephrotoxicity in patients receiving AmBd [2]. Typically, either 500 mL or 1,000 mL normal saline is prescribed as a pre-infusion before each AmBd dose [3,4,5,6,7]. This one-size-fits-all approach would translate to the different doses of the normal saline pre-infusion relative to the body weight or the amount of AmBd. We hypothesized that the different doses of the normal saline pre-infusion received might result in different effectiveness. This report examined the effect of different normal saline pre-infusion doses, and other potential risk factors, on the development of acute kidney injury (AKI) in patients with invasive fungal infection receiving AmBd.

## Methods

Adult patients (age ≥15 years old) with invasive fungal infections who received intravenous AmBd at our center from January 2012 through December 2014 were eligible for inclusion in this retrospective study. Patients were excluded from the study if they were admitted to the intensive care units, received vasoactive drugs, or had a baseline serum creatinine of more than 2 mg/dL.

Ethical approval of this study was obtained from the institutional review board. The written informed consent was obtained. Patients with invasive fungal infection during the study period were identified from the electronic diagnosis database. Patients who received intravenous AmBd and did not have the exclusion criteria were included in the study. Clinical and laboratory data of the included patients during the AmBd therapy were then extracted from medical records.

AmBd was typically dissolved in 5% dextrose in water (up to 0.1 mg/mL) and given intravenously over 2–6 h. Premedications with intravenous chlorpheniramine (10 mg) and oral acetaminophen (500–1,000 mg) were administered before each AmBd dose. Normal saline pre-infusion, either 500 mL or 1,000 mL, was given over 2–4 h. Dietary intake, fluid intake, and additional fluid therapy (if needed) were prescribed according to the usual practice.

The outcome of interest was AmBd-associated AKI, defined as an increase in serum creatinine by at least 0.3 mg/dL within 48 h, or at least 50% of baseline within 7 d [8]. The outcome was observed until the AmBd was stopped, temporarily withheld, or switched to other amphotericin B formulations.

Categorical variables were expressed as number (percentage), while continuous variables were expressed as mean ± standard deviation (SD) or median (interquartile range) if their distributions were skewed. Doses of the normal saline pre-infusion were adjusted to the body weight (mL/kg BW) and the daily dose of amphotericin B (mL/mg AmBd).

Kaplan–Meier survival analysis was used to estimate 14 d AKI-free survival rates, and the log-rank test was used to compare AKI-free survivals between groups. Continuous variables were converted into quartiles, and AKI-free survivals among the quartiles were compared using the log-rank test for trend. The effects of covariates on the association between risk factors and AKI-free survival were examined by stratified log-rank test. Cox proportional hazard model was not appropriate for multivariate analysis in this study because risk factors identified by the log-rank test did not meet the proportional hazard assumption.

## Results

The study encompassed 60 patients, with 31 patients developing AKI during AmBd therapy. Characteristics of the participants are summarized in **Table 1**.

**Table 1.** Characteristics of the participants

**Characteristics**	**Participants (n = 60)**
Age (years)	40.3 ± 13.8
Female gender	27 (45.0%)
Comorbidity	
HIV infection	41 (68.3%)
Hematologic malignancies	10 (16.7%)
Diabetes mellitus	2 (3.3%)
Fungal infection	
Cryptococcosis	46 (76.7%)
Aspergillosis	14 (23.3%)
Concomitant use of nephrotoxic drugs	
Amikacin	2 (3.3%)
Vancomycin	5 (8.3%)
Tenofovir disoproxil fumarate	4 (6.7%)
Body weight (kg)	50.3 ± 10.8
Dose of AmBd (mg/kg/d)	0.90 ± 0.15
Duration of AmBd therapy (d)	14 (7–20)
Dose of NSS pre-infusion	
(a) Adjusted to body weight, NSS/BW (mL/kg BW)	10.5 ± 2.0
(b) Adjusted to AmBd dose, NSS/AmBd (mL/mg AmBd)	11.9 ± 3.0
Baseline BUN (mg/dL)	12.8 ± 5.3
Baseline Cr (mg/dL)	0.77 ± 0.21
Baseline CKD-EPI eGFR (mL/min/1.73 m^2^)	106.3 ± 23.1
Baseline BUN/Cr ratio	17.3 ± 7.4
Baseline sodium (mEq/L)	132.5 ± 5.6
Baseline potassium (mEq/L)	3.90 ± 0.43
Baseline chloride (mEq/L)	99.0 ± 5.7
Baseline bicarbonate (mEq/L)	22.6 ± 3.6
Baseline magnesium (mg/dL), incomplete data (n = 40)	0.79 ± 0.14
Acute kidney injury	31 (51.7%)

Data are presented as mean ± SD, median (interquartile range), or number (percent).

AmBd, amphotericin B deoxycholate; BUN, baseline blood urea nitrogen; NSS, normal saline solution; SD, standard deviation.

The 500 mL normal saline pre-infusion was administered to 59 patients, and the 1,000 mL normal saline pre-infusion to 1 patient. The dose of normal saline pre-infusion adjusted to the body weight (NSS/BW) was 10.5 ± 2.0 mL/kg BW, while the dose adjusted to the amount of AmBd given daily (NSS/AmBd) was 11.9 ± 3.0 mL/mg AmBd.

**Figure 1.**
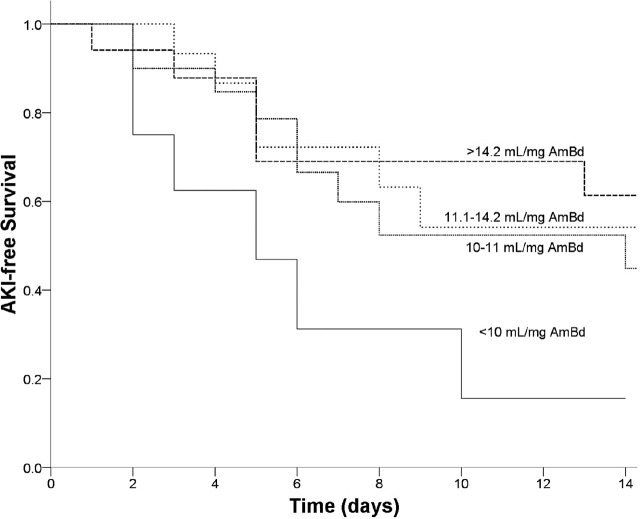
Kaplan–Meier curve of AKI-free survival for each quartile of NSS/AmBd (*P* = 0.03 by log-rank test for trend). AKI, acute kidney injury; NSS/AmBd, dose of normal saline pre-infusion adjusted to amphotericin B dose.

The overall 14 d AKI-free survival was 48.3%. NSS/AmBd, but not NSS/BW, was associated with AKI-free survival (**Table 2**). Higher NSS/AmBd was associated with higher AKI-free survival (**Figure 1** and **Table 3**). The patients who had NSS/AmBd in the lowest quartile (<10 mL/mg AmBd) had lower AKI-free survival than those who had NSS/AmBd in the other quartiles. The patients receiving less than 10 mL/mg AmBd of normal saline pre-infusion had significantly lower AKI-free survival than those receiving 10 mL/mg AmBd or more (*P* = 0.02; 14 d AKI-free survival 15.6% vs. 53.3%, respectively).

**Table 2.** Univariate analysis to identify factors associated with AKI-free survival using the log-rank test and the log-rank test for trend

**Characteristics**	**Median AKI-free survival in each subgroup (d)**	***P***
Age (quartile)	>32, >35, 13, 14	0.65
Gender (male vs. female)	8 vs. >32	0.01
HIV infection (yes vs. no)	14 vs. >21	0.79
Hematologic malignancies (yes vs. no)	5 vs. 16	0.21
Diabetes mellitus (yes vs. no)	10 vs. 14	0.51
Cryptococcosis (yes vs. no)	16 vs. 5	0.11
Concomitant nephrotoxics (yes vs. no)	16 vs. 13	0.78
Body weight (quartile)	17, >35, 14, 5	0.06
Dose of AmBd (quartile)	>21, 10, 16, 5	0.40
NSS/BW (quartile)	6, 14, >35, 17	0.09
NSS/AmBd (quartile)	5, 14, 17, >21	0.03
Baseline BUN (quartile)	8, 6, 14, >35	0.04
Baseline Cr (quartile)	14, 10, 7, 18	0.87
Baseline CKD-EPI eGFR (quartile)	18, 20, 13, 6	0.17
Baseline BUN/Cr ratio (quartile)	5, 7, 16, 18	0.09
Baseline sodium (quartile)	16, 8, >21, 6	0.25
Baseline potassium (quartile)	13, 16, >35, 8	0.45
Baseline chloride (quartile)	6, 13, 9, 17	0.35
Baseline bicarbonate (quartile)	18, 14, 5, 6	0.04
Baseline magnesium (quartile)	10, 18, 13, 6	0.67

AKI, acute kidney injury; AmBd, amphotericin B deoxycholate; BUN, baseline blood urea nitrogen; NSS/AmBd, dose of normal saline pre-infusion adjusted to amphotericin B dose; NSS/BW, dose of normal saline pre-infusion adjusted to body weight.

**Table 3.** The 14 d AKI-free survival rate in each group/quartile of the significant risk factors

**Particulars**	**14 d AKI-free survival rate (percentage without AKI)**
Gender	
Male	37.2
Female	63.1

NSS/AmBd (mL/mg)	
<10^†^	15.6
10–11	44.9
11.1–14.2	54.2
>14.2^†^	61.4

Baseline BUN (mg/dL)	
<9	36.4
9–12^‡^	29.4
13–15	47.6
>15^‡^	73.4

Baseline bicarbonate (mEq/L)	
<21^§^	65.7
21–22	47.3
23–24^§^	34.1
>24	40.7

† Had different AKI-free survival in pairwise log-rank test, *P* = 0.03.

‡ Had different AKI-free survival in pairwise log-rank test, *P* = 0.01.

§ Had different AKI-free survival in pairwise log-rank test, *P* = 0.03.

AKI, acute kidney injury; AmBd, amphotericin B deoxycholate; BUN, baseline blood urea nitrogen.

Gender, baseline blood urea nitrogen (BUN), and baseline plasma bicarbonate (Bicarb) were also associated with AKI-free survival. The other factors (age, comorbidities, body weight, dose of amphotericin B, baseline plasma creatinine, baseline CKD-EPI eGFR, baseline BUN/Cr ratio, baseline plasma sodium, baseline plasma potassium, baseline plasma chloride, and baseline plasma magnesium) were not associated with AKI-free survival (**Table 2**). Female gender, higher BUN, and lower Bicarb were associated with higher AKI-free survival (**Table 3**).

Among the 4 risk factors for AmBd-associated AKI identified in the present study, only NSS/AmBd and BUN were likely to be the independent risk factors (**Table 4**). NSS/AmBd was still associated with AKI-free survival in the gender-stratified and BUN-stratified, as well as Bicarb-stratified, log-rank test for trend. BUN was also associated with AKI-free survival in the gender-stratified and NSS/AmBd-stratified, as well as Bicarb-stratified, log-rank test for trend.

**Table 4.** Effect of the significant covariates on association between risk factors and AKI-free survival examined by the stratified log-rank test and the stratified log-rank test for trend

**Risk factors**	***P* value for association between risk factors and AKI-free survival**			

	**Gender-stratified**	**NSS/AmBd-stratified**	**BUN-stratified**	**Bicarb-stratified**
Gender	–	0.02	0.07	0.08
NSS/AmBd (quartile)	0.04	–	0.03	0.02
BUN (quartile)	0.02	0.01	–	0.04
Bicarb (quartile)	0.18	0.04	0.36	–

AKI, acute kidney injury; Bicarb, baseline plasma bicarbonate; BUN, baseline blood urea nitrogen; NSS/AmBd, dose of normal saline pre-infusion adjusted to amphotericin B dose.

## Discussion

Patients receiving AmBd are at risk for developing AKI from renal vasoconstriction [9]. Volume expansion with salt loading has been shown to effectively reduce the AmBd-associated AKI by decreasing renal vasoconstriction [10].

Currently, normal saline pre-infusion 500–1,000 mL is recommended in all patients receiving AmBd [2]. Our data suggest that the normal saline pre-infusion at doses lower than 10 mL/mg AmBd might not be as effective as at the higher doses. Therefore, the 500 mL normal saline pre-infusion might be a reasonable pre-infusion regimen for patients receiving AmBd up to 50 mg/d, but not for those receiving the higher daily dose of AmBd. For those receiving AmBd more than 50 mg/d, the 1,000 mL normal saline loading is preferred, and at least 10 mL/mg AmBd of the normal saline should be given as the pre-infusion.

A higher dose of AmBd might induce a higher degree of renal vasoconstriction. Consequently, more sodium loading is required to prevent renal vasoconstriction. Therefore, customizing the amount of salt loading according to the amount of AmBd given is a reasonable practice. A salt-loading regimen that used this approach (giving more sodium chloride to the patients receiving a higher dose of AmBd) resulted in cumulatively 1,291 d of AmBd treatment without renal failure [11].

The present study also identified additional factors associated with AmBd-associated AKI: gender, BUN, and Bicarb. Our finding confirms the result of the previous reports that males may have a higher risk of AmBd-associated AKI than females [12, 13]. The mechanism for this gender gap is unclear.

The association between the lower BUN and the higher risk of AmBd-associated AKI found in the present study is rather unexpected but not unprecedented. In a previous report, patients with AmBd-associated nephrotoxicity marginally had lower BUN than patients without AmBd-associated nephrotoxicity [14].

Since Cr and eGFR did not associate with AmBd-associated AKI in the present study, the association between BUN and AmBd-associated AKI is unlikely to be related to renal function. One plausible explanation for this association is dietary protein-induced renal vasodilatation. Patients with low protein intake would have low BUN and low levels of dietary protein-induced renal vasodilatation [15]. This physiologic change put the patients at risk for the vasoconstrictive AmBd-associated AKI. Whether increasing protein intake could prevent AmBd-associated AKI should be further explored, preferably by a randomized controlled trial. In the meantime, it is reasonable to avoid inadequate protein intake in the patients receiving AmBd.

The connection between the higher Bicarb and the higher risk of AmBd-associated AKI demonstrated in the present study is probably related to chloride depletion. Chloride depletion is associated with metabolic alkalosis and decreased renal bicarbonate excretion [16]. Patients with pre-existing chloride depletion would have high Bicarb and are more susceptible to the AmBd-associated AKI. Correction of pre-existing chloride depletion before initiating AmBd therapy could not be overemphasized.

The associations identified from the present retrospective study are informative but far from definitive. Because the formal multivariable analysis was not carried out, none of the risk factors for AmBd-associated AKI identified in the present study could be declared independent risk factors. Further investigations regarding these risk factors and corresponding preventive measures for AmBd-associated AKI are warranted.

## Conclusions

The present study suggests that an insufficient amount of normal saline pre-infusion relative to the amount of AmBd given daily (NSS/AmBd), male gender, low BUN, and high Bicarb are risk factors for AmBd-associated AKI. Excluding gender, these risk factors are potentially modifiable and would guide tailoring appropriate preventive measures for AmBd-associated AKI.
